# Supplementary material for: Pricing indirect emissions accelerates low—carbon transition of US light vehicle sector
Source: Nat Commun. 2021 Dec 8;12:7121. doi: 10.1038/s41467-021-27247-y (PMC8654946; doi:10.1038/s41467-021-27247-y)
Supplement: Supplementary file 1 — Editor Summary [file 41467_2021_27247_MOESM1_ESM.docx]

New research shows how large-scale adoption of electric vehicles due to expected technological change may not only reduce emissions from tailpipes, but also indirect emissions stemming from energy and battery production.
