# Supplementary material for: Pricing indirect emissions accelerates low—carbon transition of US light vehicle sector
Source: Nat Commun. 2021 Dec 8;12:7121. doi: 10.1038/s41467-021-27247-y (PMC8654946; doi:10.1038/s41467-021-27247-y)
Supplement: Supplementary file 2 — Supplementary Information [file 41467_2021_27247_MOESM2_ESM.pdf]

Supplementary Information

Pricing indirect emissions accelerates low–carbon transition of US light vehicle sector

Paul Wolfram[1*], Stephanie Weber[1], Kenneth Gillingham[1,2], and Edgar Hertwich[1,3]

[1]Yale University, School of the Environment
[2]Yale University, School of Management
[3]Industrial Ecology Programme, Department of Energy and Process Engineering, Norwegian University of Science and Technology
[*]E-mail: paul.wolfram@aya.yale.edu

Contents

1 Brief summary **3**

2 Specific vehicle life–cycle emissions **3**

3 Graphical model description **6**

4 Technology costs and vehicle choice **7**

5 Full list of analyzed scenarios **10**

6 Potential emission reductions due to reuse and recycling **11**

7 Detailed results of the uncertainty analysis **12**

7.1 Uncertainty in vehicle sales and life–cycle emissions due to costs of batteries and renewables 12

7.2 Uncertainty in vehicle sales due to battery capacities 14

8 Vehicle fleet characteristics **15**

9 Electricity supply assumptions for the main scenarios and side cases **16**

10 Defining 'integrated energy model' **17**

11 Additional notes on vehicle materials **17**

List of Figures

1 Specific life–cycle emissions of modelled powertrains in all scenarios 4
2 Specific life–cycle emissions broken down by life–cycle stage in 2010 and 2050 5
3 Graphical representation of soft–linking a life cycle assessment model to Yale–NEMS. 6
4 Purchase price development of selected powertrains in the midsize car segment 7
5 Feebate (in USD/vehicle) on vehicle production (a), energy carrier production (b), total (c), on energy carrier production assuming low–carbon hydrogen (d) 8
6 Optimal vehicle choice in the side cases under full pricing (a), and well–to–wheel (WTW) pricing (b, c) . . 9
7 Life–cycle CO_2 emissions of producing the stock of US light vehicles 11
8 Optimal vehicle choice under direct–emissions–only pricing (‘Direct–only’) (a–c), and full emissions pricing (‘Full’) (d–f) and assuming constant costs of EV batteries (‘High–$ EV’) and/or renewable electricity generators (‘High–$ RE’). 12
9 Life–cycle CO_2 emissions of the US light vehicle fleet, total (a) and broken down by life–cycle stage (b–e) when fully pricing emissions (‘Full’) and when only pricing direct emissions (‘Direct’) and assuming constant costs of EV batteries (‘High–$ EV’) and/or renewable electricity generators (‘High–$ RE’). 13
10 Optimal vehicle choice under direct–emissions–only pricing (‘Direct’) (a,c), and full emissions pricing (‘Full’) (b,d) and assuming improving battery densities (‘dens’) (c,d). 14
11 Characteristics of the US light vehicle fleet under full–emissions pricing and direct–emissions–only pricing . 15
12 Total electricity generated by utility companies, residential and commercial buildings in all scenarios other than the sensitivity cases 16
13 CO_2 emissions and emissions intensity from total electricity generated by utility companies, residential and commercial buildings (a) and development of gasoline prices and electricity prices in all scenarios other than the sensitivity cases (b). Energy prices in US cents per British thermal unit (c) 16
14 Total power plant capacity additions by utility companies, residential and commercial buildings (a) and power plant capacity retirements by utility companies in all scenarios other than the sensitivity cases (b) 17

1 Brief summary

A large–scale transition towards electric vehicles can greatly reduce emissions from vehicle tailpipes. However, there is great concern that it can come with increased indirect emissions from electricity and battery production that are not commonly regulated by transport policies. Indeed, neither model calculations, nor real–world policies, have fully accounted for or priced indirect vehicle emissions to date. Here, we propose an interdisciplinary approach by combining integrated energy modeling and industrial ecology methods. With it we compare optimal policy scenarios that price emissions at the tailpipe only, versus both tailpipe and indirect emissions. Surprisingly, scenarios that also price indirect emissions exhibit higher, rather than reduced, sales of electric vehicles. Simultaneously, these scenarios of higher electric vehicle adoption, not only yield lower cumulative tailpipe emissions but also lower indirect emissions. Expected technological change ensures that emissions from electricity and battery production are more than offset by reduced emissions of gasoline production. Material efficiency measures, such as recycling of materials and reuse of vehicle components have the potential to further offset increased emissions from batteries. Given continued decarbonization of electricity supply, results show that a large–scale adoption of electric vehicles is able to reduce CO_2 emissions through more channels than previously expected. Further, carbon pricing of stationary sources will also favor electric vehicles. The results shed new light on the current public debate about ‘dirty’ batteries and electricity. In fact, the simultaneous reduction of both direct and indirect emissions indicates a win–win situation for climate change mitigation, meaning that climate policy with very high shares of electric vehicles represents a no–regrets strategy (but only if electricity continues to decarbonize as has been assumed in our main scenarios). Our insights are therefore highly relevant for global climate and transport policies. Current policies, such as performance standards or emission pricing schemes, should be broadened in their scope in order to regulate all sources of vehicle emissions along the entire supply chain or throughout the entire life cycle. Our scenarios further indicate that the US (and likely other nations with suitable low–carbon electricity grids) should target deployment of fully electric vehicles. Our work represents a step towards a holistic inclusion of dynamic life–cycle relationships in integrated modelling frameworks. Future research could include additional potentially important factors and processes as well as investigate the degree to which our results would differ in various regions of the world, or if additional pollutants, other than direct and indirect CO_2 emissions, were internalized in optimal pollution mitigation pathways.

2 Specific vehicle life–cycle emissions

Specific life–cycle emissions in g CO_2/km include all life stages including the entire vehicle supply chain (considering material production, vehicle assembly, recycling of materials, reuse of components, and material substitution through lightweighting), as well as the entire energy chain (production and use of energy carriers). A detailed description of the specific production processes can be found elsewhere [1, 2] and all assumed data points are provided in an accompanying spreadsheet file. Here we calibrate these processes to the US case as described in the Methods section in the main text.

It can be seen from Supplementary Figure 1 that fossil–fueled vehicles remain at more or less steady specific emissions after 2025, which is the last year of the CAFE requirement in Yale–NEMS. Emissions for producing gasoline are assumed at 70 g CO_2/kWh or ∼2,300 g CO_2/gallon gasoline equivalents (GGE) [3, 4], compared to direct emissions (synonymous with tailpipe emissions) of about 250 g CO_2/kWh or ∼8,300 g CO_2/GGE. Specific emissions of BEVs, and of PHEVs to some extent, continue to fall due to decarbonization of the electric grid. In a side case we assume that hydrogen production transitions from the currently assumed steam methane reforming (SMR) pathway to an illustrative net–zero emissions pathway. Net–zero could be achieved in various different ways, for example by producing hydrogen exclusively from wind or solar power or by using a hydrogen production mix consisting primarily of hydrogen from biomethane with carbon capture and storage, representing a carbon sink with about – 36 g CO_2e/kWh [5], and a small remainder, around 7–8%, of hydrogen from SMR, emitting around 450 g CO_2e/kWh [6]. Since carbon–neutral hydrogen production is only considered in one of our side cases it has been modeled in less detail: For the carbon–neutral hydrogen side case NEMS only receives the hydrogen production emissions factor from the LCA model, which can be assumed equal under both production pathways. Hence, for the purpose of this paper, whether hydrogen is produced from renewables or from a combination of biomethane CCS and natural gas, has no effect on the emissions outcomes because both production pathways would ensure net–zero emissions.

Supplementary Figure 2 provides a breakdown of specific emissions by life–cycle stage. Compared to previous work [7, 8, 1] emissions embodied in vehicle production are relatively small when normalized by vehicle kilometers travelled (Supplementary Figure 2d, e). This is mostly due to the relatively high average lifetime mileage of US light vehicles of around 270,000 km [9] which is reflected in Yale–NEMS. Previous work has often assumed kilometers travelled in the range of 150,000–180,000 over the lifetime of a vehicle [7, 8, 1]. The GHG intensity of batteries in particular (and vehicle production in general) improves in several ways in our model (Supplementary Figure 2d, e). The main scenarios assume a falling

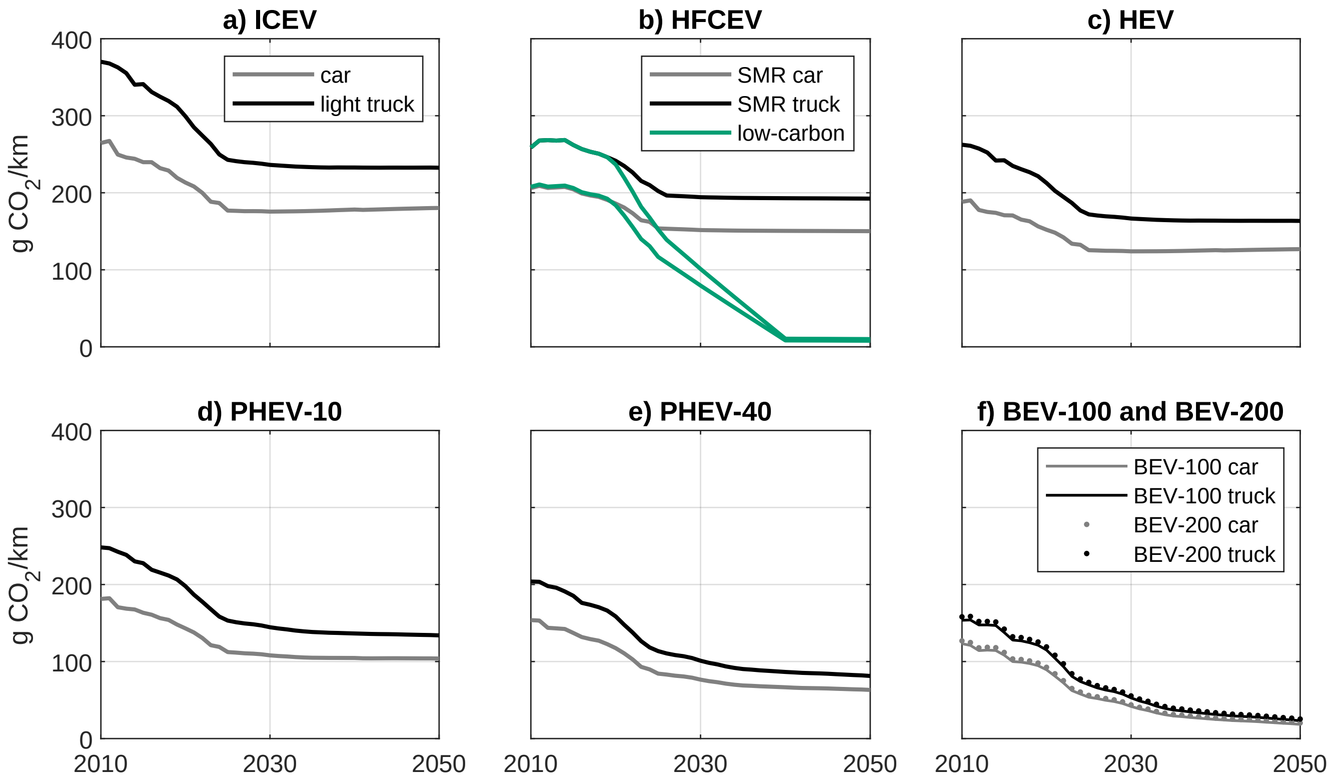

Supplementary Figure 1: Specific life–cycle emissions of modelled powertrains in the main scenarios and side cases. ICEV=internal combustion engine vehicle; HEV=hybrid electric vehicle; PHEV=plug–in hybrid electric vehicle; BEV=battery electric vehicle; HFCEV=hydrogen fuel cell electric vehicle; –10=10 miles electric range; SMR=steam–methane reforming. The underlying data used to compile this figure can be found in Supplementary Table 1.

carbon intensity of electricity, which reduces both the emissions during the material production stage as well as emissions invoked during the battery assembly stage (Supplementary Table 18). The assumed energy mix of the material production and battery assembly stages is comprised of heat from fossil fuels as well as electricity (see Supplementary Tables 19, 20). In addition, some scenarios assume improved recycling of materials and reuse of components such as batteries (see Supplementary Tables 21, 22), further reducing GHG emissions.

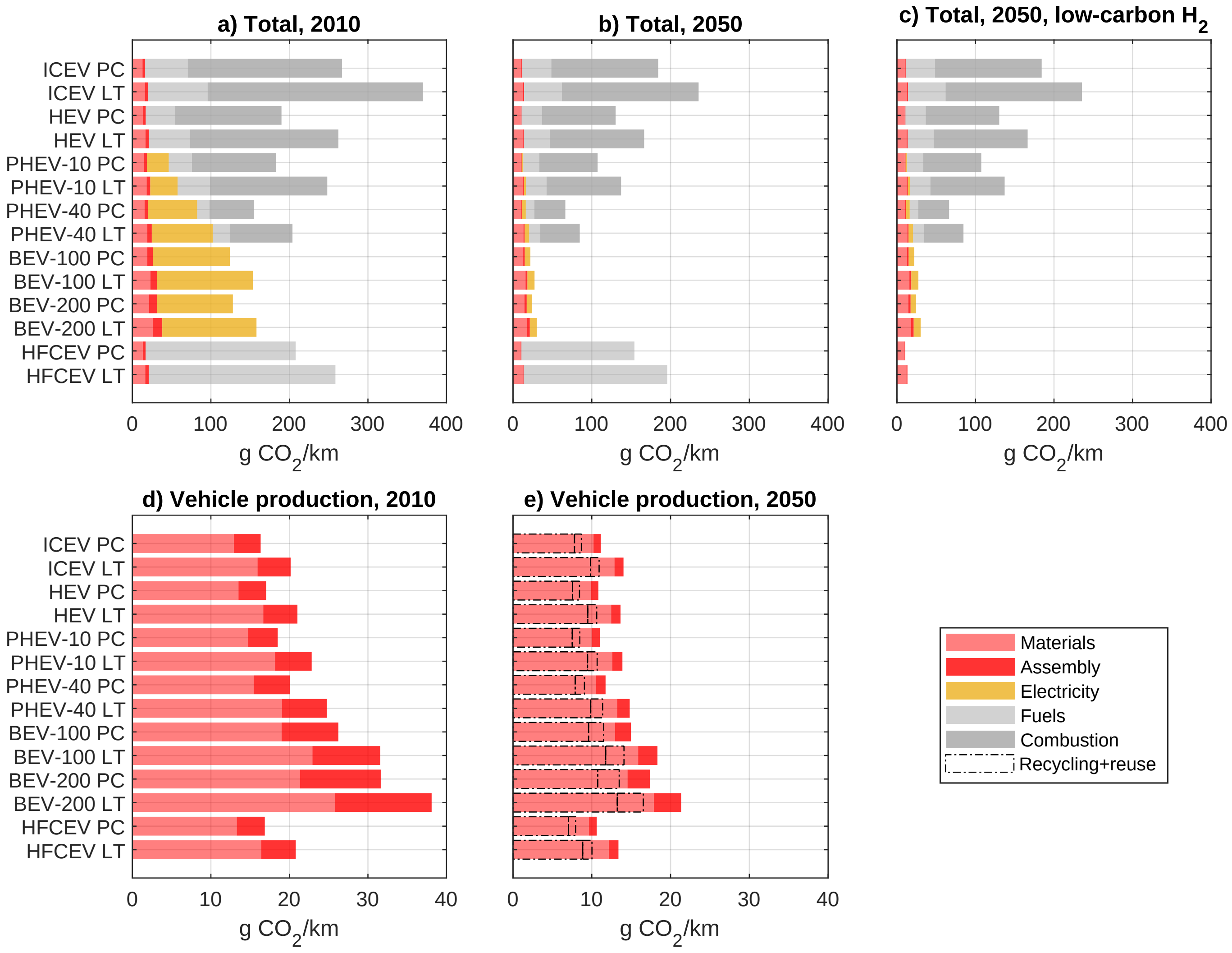

Supplementary Figure 2: Specific life–cycle emissions broken down by life–cycle stage in 2010 and 2050. ICEV=internal combustion engine vehicle; HEV=hybrid electric vehicle; PHEV=plug–in hybrid electric vehicle; BEV=battery electric vehicle; HFCEV=hydrogen fuel cell electric vehicle; –10=10 miles electric range; PC=passenger car; LT=light truck; H_2=hydrogen. The underlying data used to compile this figure can be found in Supplementary Table 1.

3 Graphical model description

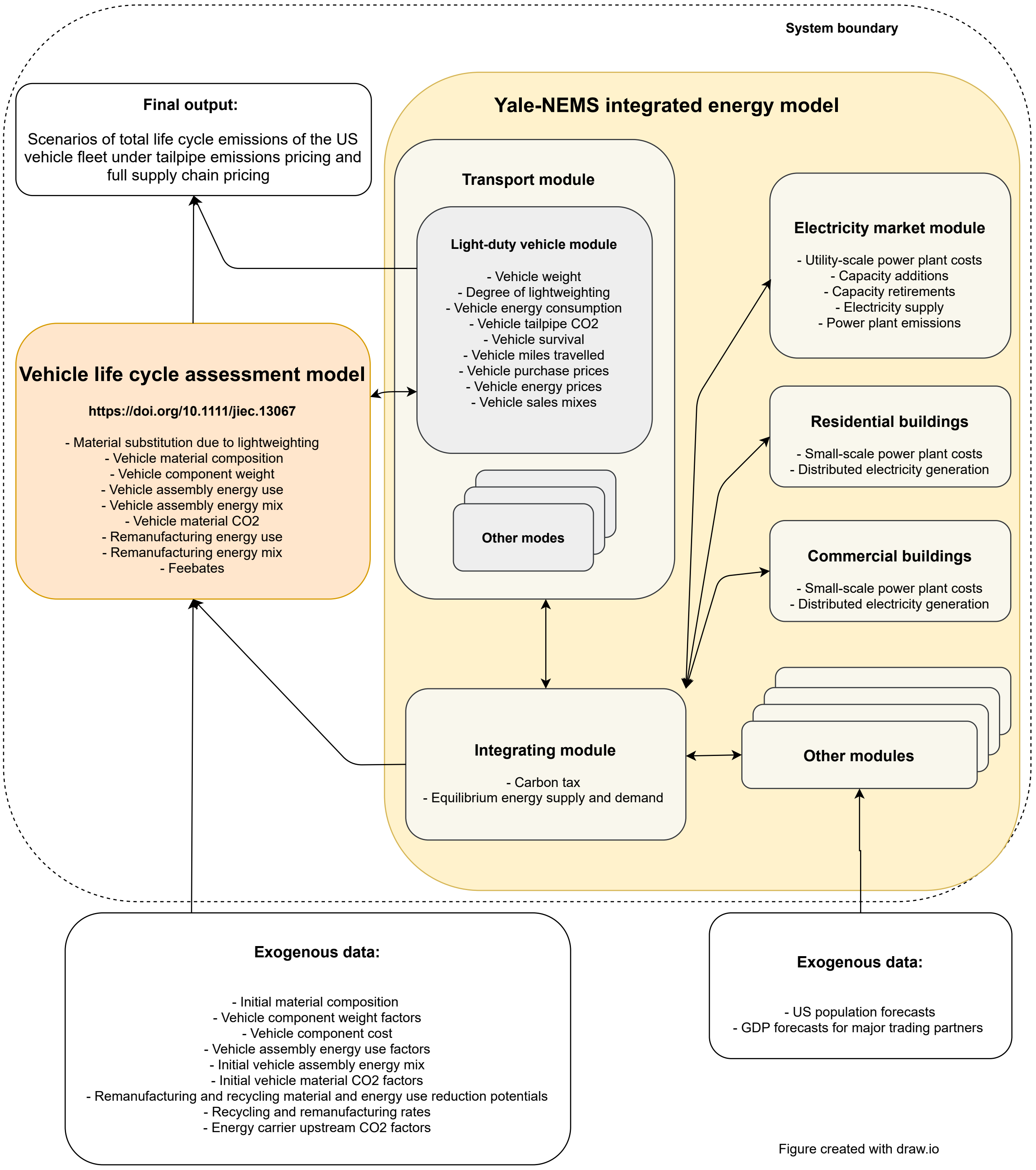

Supplementary Figure 3: Graphical representation of soft–linking a life cycle assessment model to Yale–NEMS.

4 Technology costs and vehicle choice

Production costs of various powertrains (ICEV, BEV, ...) are calculated using a detailed bottom–up cost model [10]. For this work the model has been extended to 2050 and battery prices reach floor costs of about 83 USD/kWh within the modeling time horizon. This cost development is within the range of previous estimates [11]. Following that, vehicle purchase prices are calculated endogenously in Yale–NEMS (Supplementary Figure 4). All cost figures take into account potential feebates. While no feebate is implemented under direct–emissions–only pricing (colored bars in Supplementary Figure 4), the full–pricing cases consider feebates on vehicle production AND on energy supply and use (Supplementary Figure 5a and b/d), while the well–to–wheel (WTW) pricing cases only consider feebates on energy supply and use (Supplementary Figure 5b/d). In scenario 'WTW accounting / low–c H_2 / low–\$ HFCEV' it is assumed that HFCEVs become cost–competitive with BEVs by 2035. The resulting optimal vehicle choice for each scenario can be seen in Supplementary Figure 6 (for the side cases) and in Figure 1 in the main text (for the main cases).

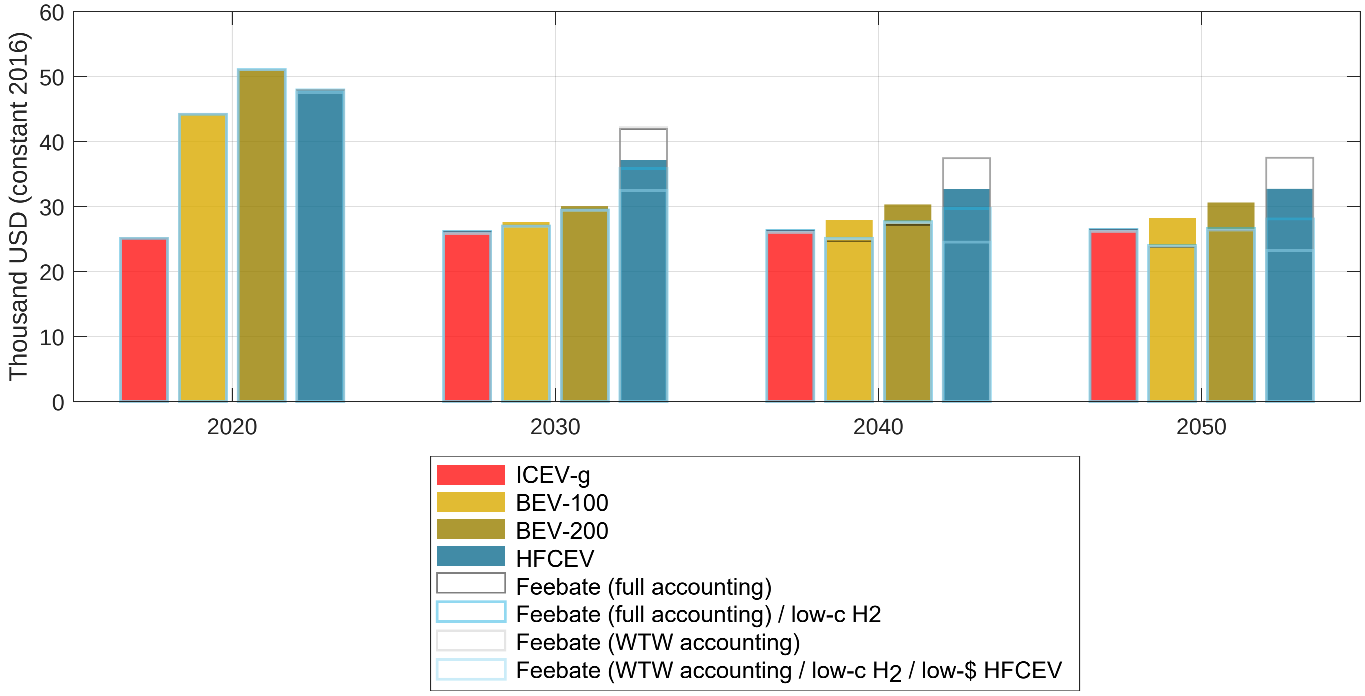

Supplementary Figure 4: Purchase price development of selected powertrains in the midsize car segment. Default figures (colored bars) show prices in the absence of feebates. WTW=well–to–wheel; low–c H_2=low–carbon hydrogen; low–\$=low–cost; ICEV=internal combustion engine vehicle; BEV=battery electric vehicle; HFCEV=hydrogen fuel cell electric vehicle; –g=gasoline; –100=100 miles of electric range; H_2=hydrogen. The underlying data used to compile this figure can be found in Supplementary Table 14.

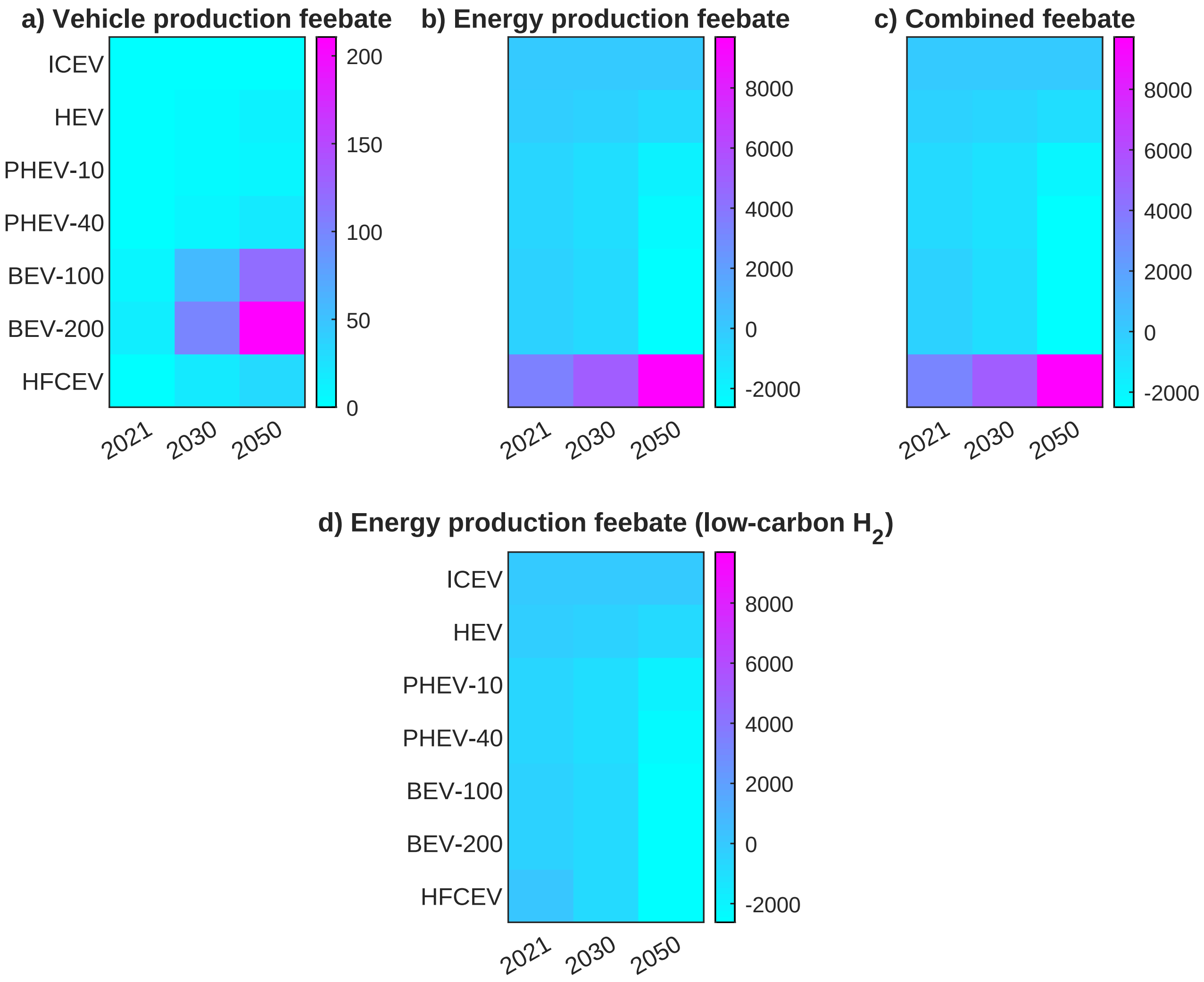

Supplementary Figure 5: Feebate (in USD/vehicle) on vehicle production (a), energy carrier production (b), total (c), and on energy carrier production assuming low–carbon hydrogen (d). ICEV=internal combustion engine vehicle; HEV=hybrid electric vehicle; PHEV=plug–in hybrid electric vehicle; BEV=battery electric vehicle; HFCEV=hydrogen fuel cell electric vehicle. –10=10 miles electric range; H_2=hydrogen. The underlying data used to compile this figure can be found in Supplementary Table 13.

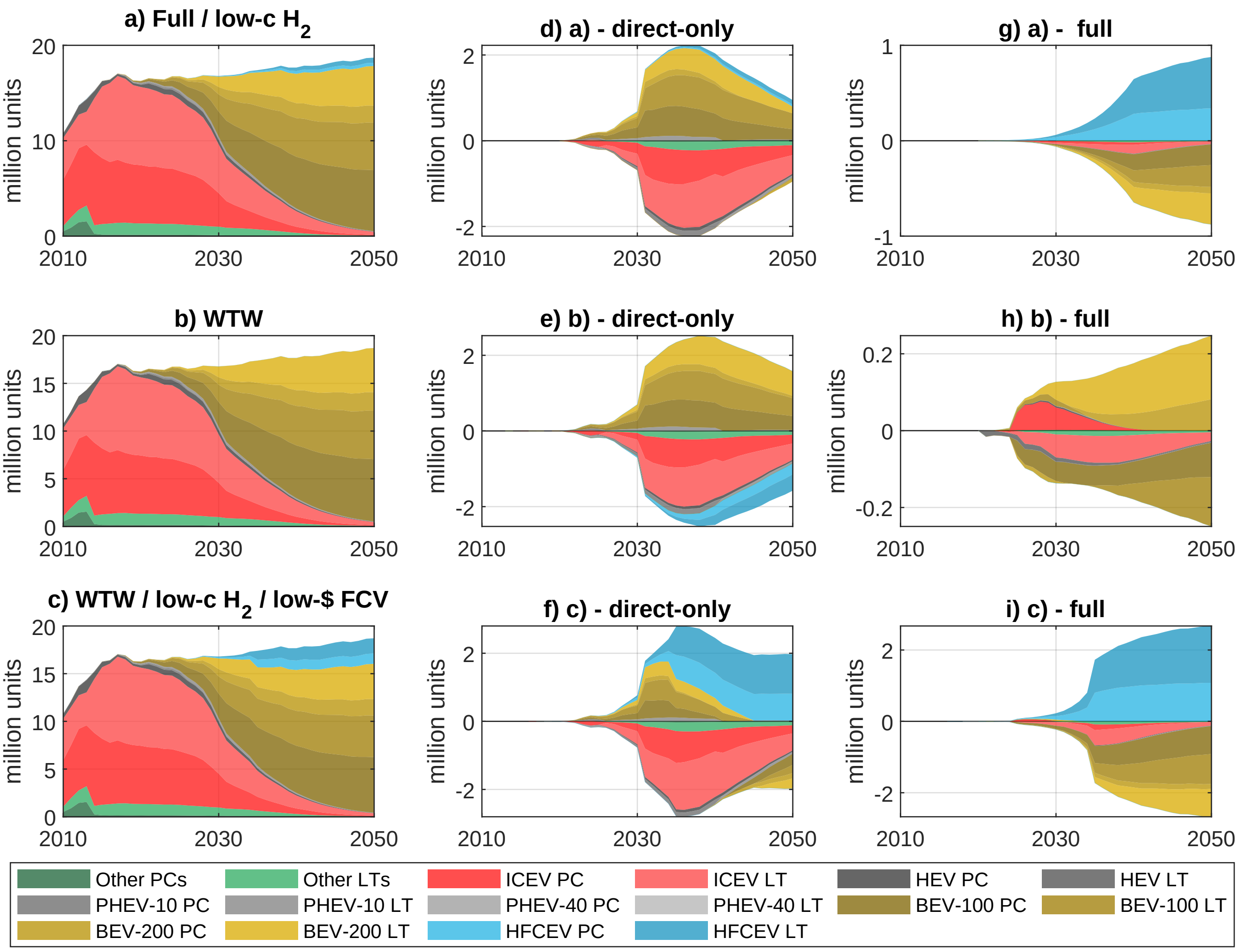

Supplementary Figure 6: Optimal vehicle choice in the side cases under full pricing (a), and well–to–wheel (WTW) pricing (b, c). A transition to low–carbon hydrogen is assumed in a) and c). Further cost reductions of HFCEVs are assumed in c). Differences between these side cases and scenario one in the main text (direct–emissions–only pricing) (d–f) and scenario two in the main text (full pricing) (g–i). low–c H_2=low–carbon hydrogen; low–$ FCV=low–cost fuel cell vehicle; ICEV=internal combustion engine vehicle; HEV=hybrid electric vehicle; PHEV=plug–in hybrid electric vehicle; BEV=battery electric vehicle; HFCEV=hydrogen fuel cell electric vehicle; –10=10 miles electric range; PC=passenger car; LT=light truck. The underlying data used to compile this figure can be found in Supplementary Table 4.

5 Full list of analyzed scenarios

1. Ref – reference case of the Annual Energy Outlook 2017
2. EV – quickly falling EV battery cost
3. RE – quickly falling renewables cost
4. EVRE – quickly falling EV battery and renewables cost
5. Ref tax – 'Ref' under tailpipe emissions pricing
6. EV tax – 'EV' under tailpipe emissions pricing
7. RE tax – 'RE' under tailpipe emissions pricing
8. EVRE tax – 'EVRE' under tailpipe emissions pricing
9. Ref tax full – 'Ref' under full supply chain emissions pricing
10. EV tax full – 'EV' under full supply chain emissions pricing
11. RE tax full – 'RE' under full supply chain emissions pricing
12. EVRE tax full – 'EVRE' under full supply chain emissions pricing
13. Ref tax full RR – 'Ref tax full' considering recycling and reuse
14. EV tax full RR – 'EV tax full' considering recycling and reuse
15. RE tax full RR – 'RE tax full' considering recycling and reuse
16. EVRE tax full RR – 'EVRE tax full' considering recycling and reuse
17. EVRE WTW tax – 'EVRE' under energy chain emissions pricing
18. EVRE WTW tax H2 – 'EVRE WTW tax' assuming carbon–neutral hydrogen
19. EVRE WTW tax H2 FC – 'EVRE WTW tax H2' assuming cost–competitive fuel cells
20. EVRE tax full H2 – 'EVRE tax full' assuming carbon–neutral hydrogen
21. FRZN EV tax – constant (frozen) costs of EV batteries and pricing of tailpipe emissions
22. FRZN EV tax full – constant costs of EV batteries and full supply chain emissions pricing
23. FRZN RE tax – constant costs of renewables and pricing of tailpipe emissions
24. FRZN RE tax full – constant costs of renewables and full supply chain emissions pricing
25. FRZN EVRE tax – constant costs of EV batteries and renewables and tailpipe emissions pricing
26. FRZN EVRE tax full – constant costs of EV batteries and renewables and full supply chain emissions pricing
27. EVRE tax dens – 'EVRE tax' and increasing EV battery densities
28. EVRE tax full dens – 'EVRE tax full' and increasing EV battery densities

6 Potential emission reductions due to reuse and recycling

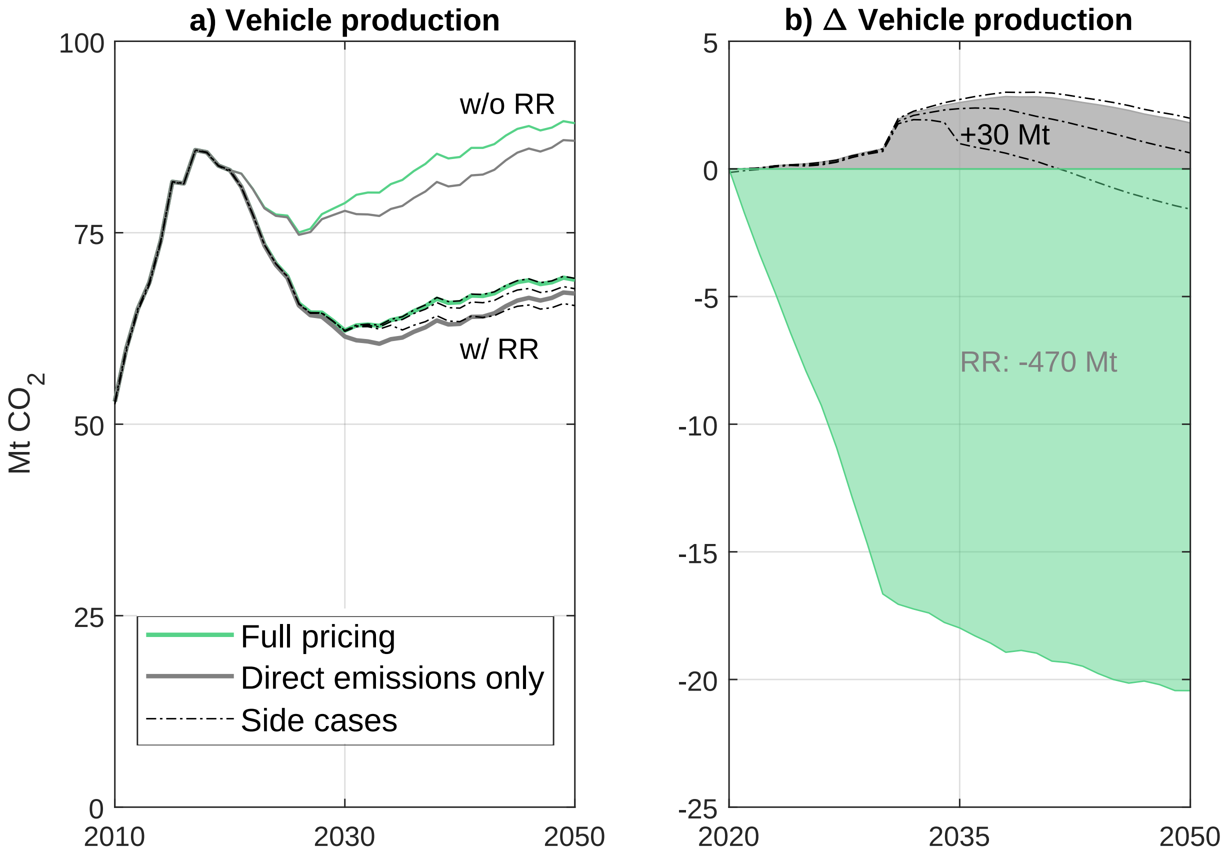

Supplementary Figure 7: Life–cycle CO_2 emissions of producing the stock of US light vehicles, under full pricing and direct–emissions–only pricing, with and without reuse and recycling (RR) (a). The grey shaded area shows the difference in emissions between full and direct–emissions–only pricing. The light green shaded area illustrates the emission reduction potential from RR (b). Dotted lines illustrate results from side cases. The underlying data used to compile this figure can be found in Supplementary Table 6.

7 Detailed results of the uncertainty analysis

7.1 Uncertainty in vehicle sales and life–cycle emissions due to costs of batteries and renewables

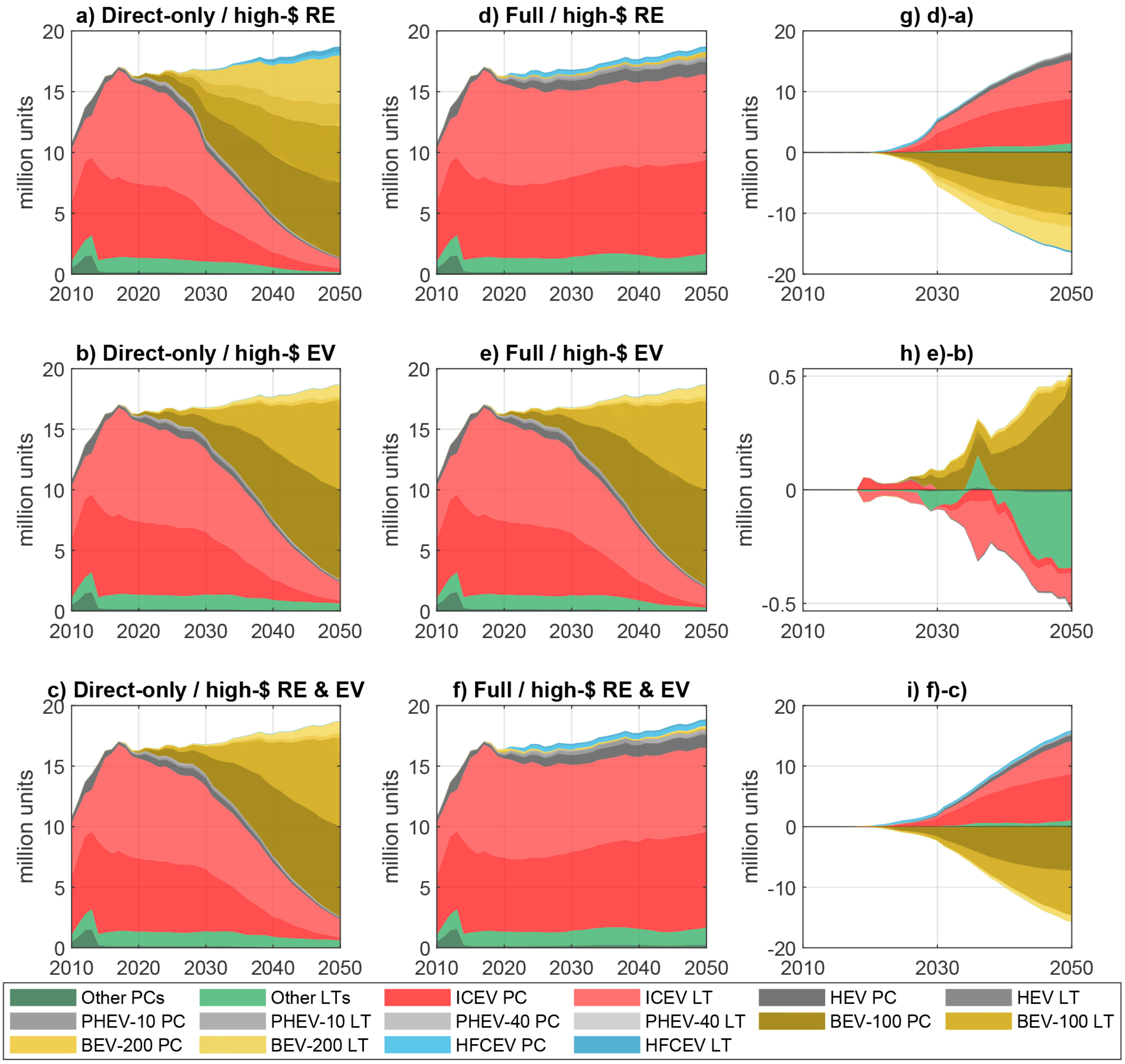

Supplementary Figure 8: Optimal vehicle choice under direct–emissions–only pricing ('Direct–only') (a–c), and full emissions pricing ('Full') (d–f) and assuming constant costs of EV batteries ('High–$ EV') and/or renewable electricity generators ('High–$ RE'). Constant costs of EV batteries are assumed in b), c), e) and f). Constant costs of renewable electricity generators are assumed in a), c), d) and f). Differences in vehicle choice between direct and full taxation (g–i). high–$ RE=constant costs of renewable electricity generators; high–$ EV=constant costs of EV batteries; ICEV=internal combustion engine vehicle; HEV=hybrid electric vehicle; PHEV=plug–in hybrid electric vehicle; BEV=battery electric vehicle; HFCEV=hydrogen fuel cell electric vehicle; –10=10 miles electric range; PC=passenger car; LT=light truck.

We assume that the cost of EV batteries remains at the 2021 level of about 200 USD/kWh which is a conservative value but within the range of recent estimates [12, 11]. Similarly, the overnight capital costs of wind and solar PV power plants remain at 2021 levels of about 1,190 USD/kW [13, 14]. As described in the main text, the electricity mix undergoes only modest changes under the assumption of constant renewables cost. In addition to a mild increase of renewables penetration, the share of natural gas grows from 32% to 43%, while that of coal falls from 25% to 13%. The average emission intensity of the fleet of natural gas power plants falls from about 475 g CO_2/kWh in 2020 to about 395 g CO_2/kWh by 2050 due to efficiency improvements and deployment of carbon capture and storage. The carbon intensity of the electricity mix falls

from about 400 g CO_2/kWh in 2020 to about 290 g CO_2/kWh by 2050. We assume the same carbon tax development as in the main cases.

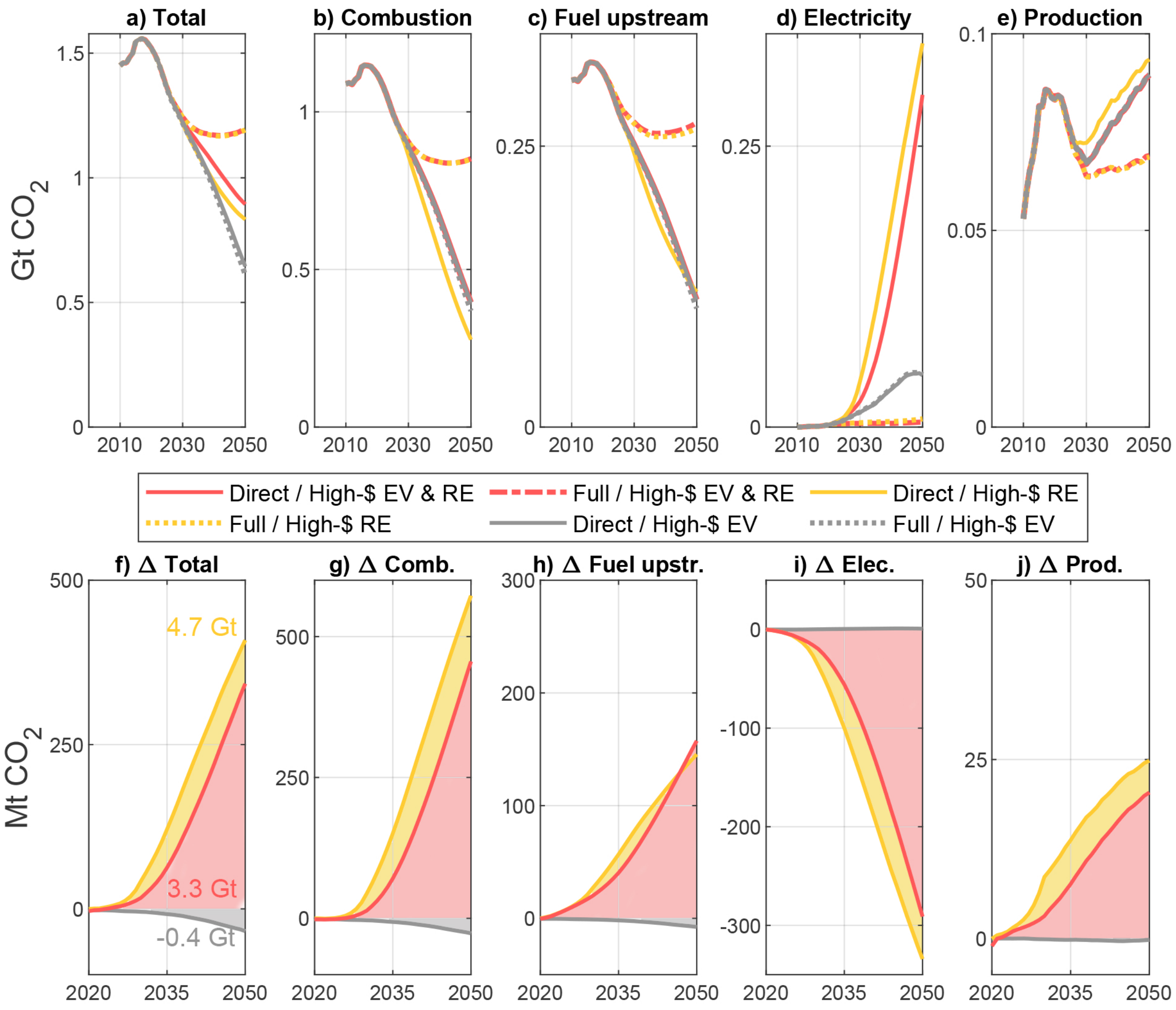

Supplementary Figure 9: Life–cycle CO_2 emissions of the US light vehicle fleet, total (a) and broken down by life–cycle stage (b–e) when fully pricing emissions (‘Full’) and when only pricing direct emissions (‘Direct’) and assuming constant costs of EV batteries (‘High–$ EV’) and/or renewable electricity generators (‘High–$ RE’). Differences in emissions between full and direct–emissions–only pricing (f–j).

7.2 Uncertainty in vehicle sales due to battery capacities

A standard assumption of Yale–NEMS is that of constant EV battery capacities (as well as constant battery weights and densities and hence EV ranges) after 2025 (Supplementary Table 11). In most of our scenario runs we adopted this assumption because, on the one hand, batteries could become more energetically dense in the future, hence requiring smaller capacities. On the other hand, larger capacities may be needed if BEVs continue to increase in driving range. Both factors could cancel each other out, hence the constant capacity assumption. However, in this section we present two sensitivity cases that explore the effects of increasing battery densities, leading to smaller, lighter, less material-intensive and cheaper EV batteries while providing the same driving range. We assume that – averaged over all technologies – battery densities continue to increase by about 1.5% per year after 2025. This rate is somewhat higher than the assumed average 2010–2025 increase of about 0.9% per year. This development helps especially with cost reductions of longer–range BEVs. This is true under both direct–emissions–only pricing and marginally more so under full–emissions pricing because the emissions penalty of the smaller batteries is also lowered. Hence, a shift in sales from 100–mile to 200–mile range BEVs can be observed. In addition, this trend is accompanied by an overall increase in BEV sales shares (Supplementary Figure 10).

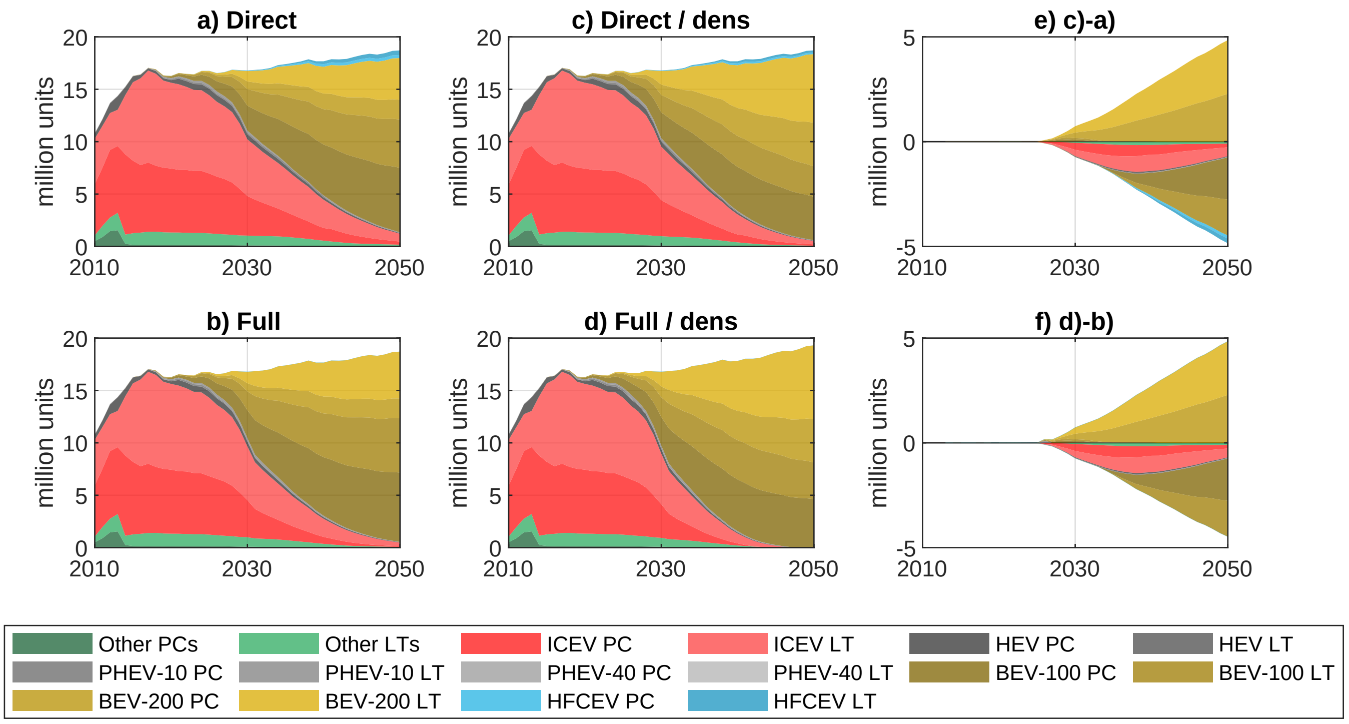

Supplementary Figure 10: Optimal vehicle choice under direct–emissions–only pricing ('Direct') (a,c), and full emissions pricing ('Full') (b,d) and assuming improving battery densities ('dens') (c,d). Differences in vehicle choice between cases with and without battery density improvements (e,f). ICEV=internal combustion engine vehicle; HEV=hybrid electric vehicle; PHEV=plug–in hybrid electric vehicle; BEV=battery electric vehicle; HFCEV=hydrogen fuel cell electric vehicle; –10=10 miles electric range; PC=passenger car; LT=light truck.

8 Vehicle fleet characteristics

While slight differences in average vehicle weight and the degree of lightweighting can be observed between the different accounting approaches (Supplementary Figure 11a, b), the curves are generally quite flat after 2025 in either scenario. This flattening can be explained by the fact that CAFE standards exist only through 2025 in Yale–NEMS. Due to the implementation of a feebate, a rebate is provided to BEVs (Supplementary Section 3), which is why average vehicle prices fall after about 2030. Conversely, vehicle prices grow considerably without a feebate system in place (Supplementary Figure 11e). Average vehicle prices are also slightly higher with greater shares of HFCEVs except in the side case in which HFCEVs reach cost parity with BEVs (Supplementary Table 14). Supplementary Figures 11d and f exhibit potential adverse side–effects of the feebate system. The lower prices seem to induce slightly lower sales shares of cars, giving rise to light trucks, while travel demand is showing marginally stronger growth. However, the differences may be too small to be of significance. The side cases exhibit some variation in these outcomes (see thin green lines in Supplementary Figures 11a–f).

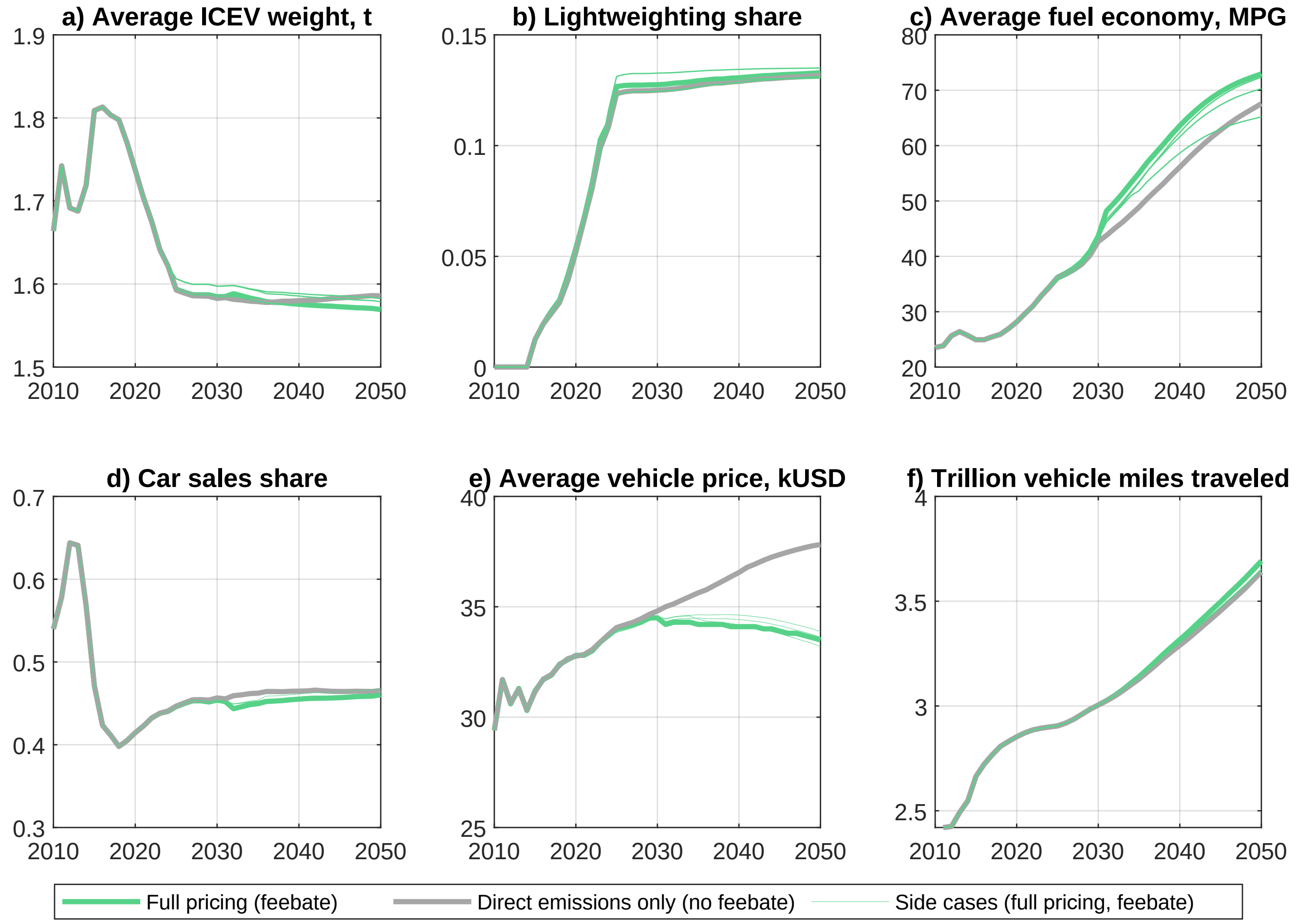

Supplementary Figure 11: Characteristics of the US light vehicle fleet under full–emissions pricing and direct–emissions–only pricing. The thin green lines show the range of results from the side cases. ICEV=internal combustion engine vehicle; MPG=miles per gallon; kUSD=thousand US dollars. Underlying data for subplots b–f can be found in Supplementary Tables 12, 5, 4, 14, 15.

9 Electricity supply assumptions for the main scenarios and side cases

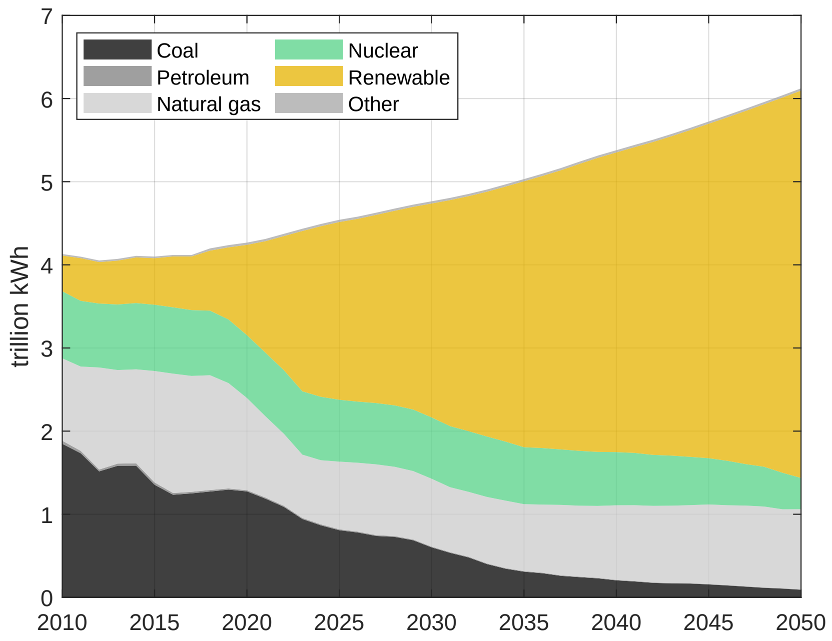

Supplementary Figure 12: Total electricity generated by utility companies, residential and commercial buildings in all scenarios other than the sensitivity cases. The underlying data used to compile this figure can be found in Supplementary Table 10.

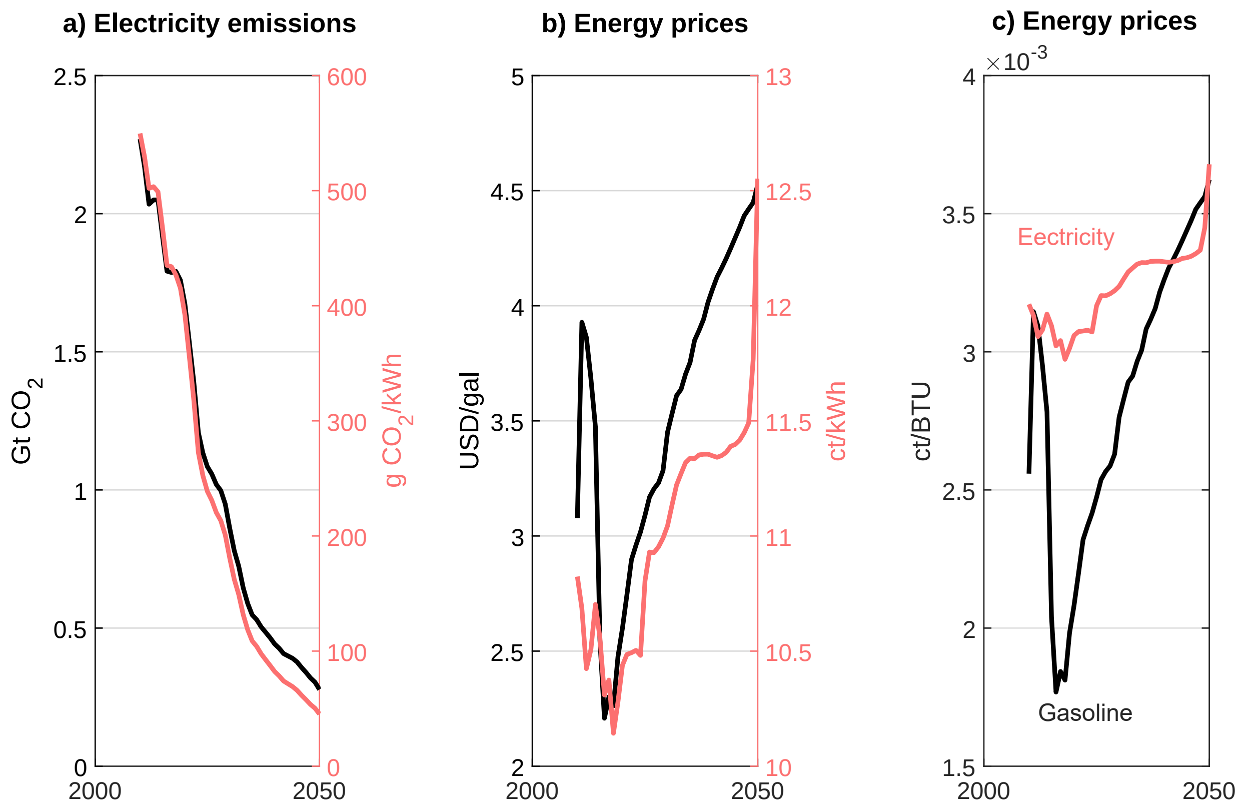

Supplementary Figure 13: CO_2 emissions and emissions intensity from total electricity generated by utility companies, residential and commercial buildings (a) and development of gasoline and electricity prices in all scenarios other than the sensitivity cases (b,c). The underlying data used to compile this figure can be found in Supplementary Tables 10, 14.

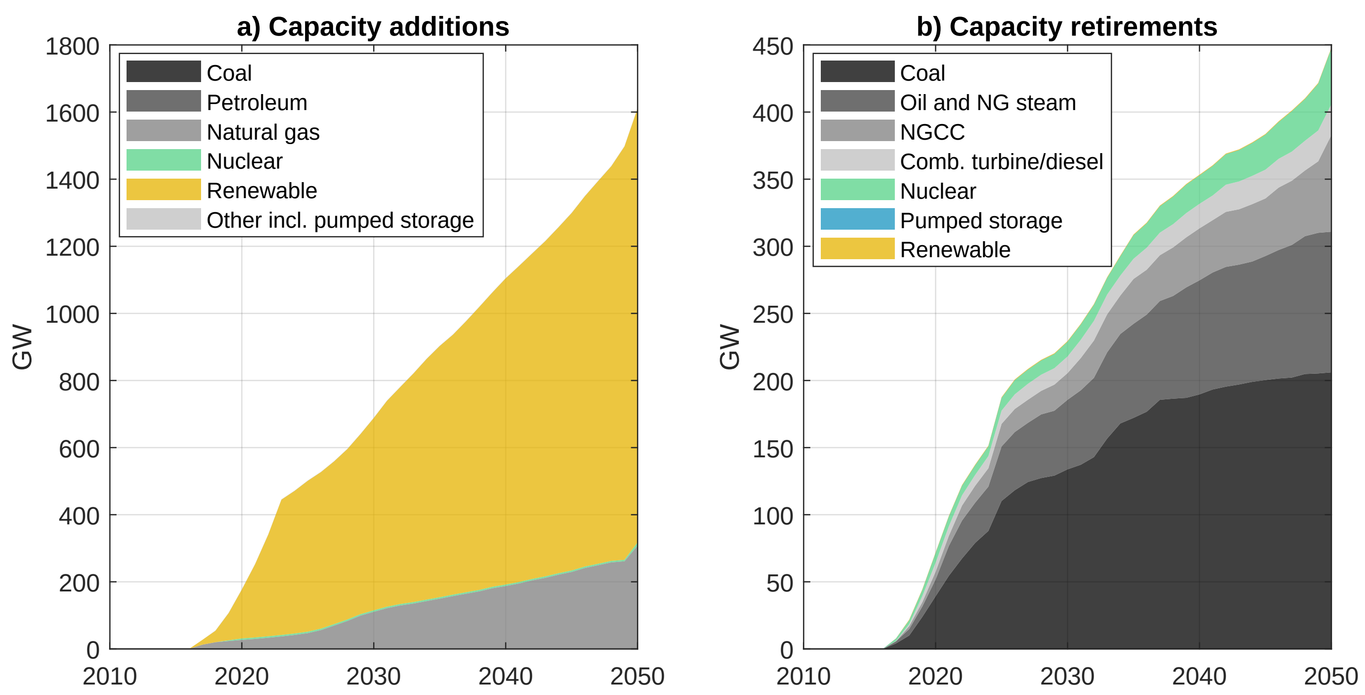

Supplementary Figure 14: Total power plant capacity additions by utility companies, residential and commercial buildings (a) and power plant capacity retirements by utility companies in all scenarios other than the sensitivity cases (b). NGCC=natural gas combined cycle. The underlying data used to compile this figure can be found in Supplementary Table 10.

10 Defining 'integrated energy model'

We define 'integrated energy models' as models that (1) entail the entire environmental–energy–economic system, (2) find an equilibrium state of energy supply and demand, either for the entire economy (general equilibrium), or for individual energy demand sectors (partial equilibrium), (3) are mathematically solved through optimization or simulation, (4) provide explicit representation of single energy demand sectors and climate change mitigation technologies within these sectors, and (5) allow exploration of future energy and emissions scenarios under different policy assumptions [15].

11 Additional notes on vehicle materials

Our detailed process–based model allows for an explicit differentiation in composition and mass of the glider and power train [1]. We consider the seven most common materials used for vehicle production: cast iron, stainless steel, automotive steel, wrought aluminum, cast aluminum, copper, and plastics (Supplementary Table 16). Combined these contribute more than 92% of the weight of the vehicle. The 'other' materials category is mostly comprised of glass and rubber [16], and for reasons of simplicity, we estimate the emissions of these other minor materials at 2 kg CO_2/kg material (Supplementary Table 17). This is well within the range of emission factors of rubber and glass in the ecoinvent 3.5 database [17]. For example, using IPCC's 2013 GWP–100 indicator, the production of synthetic rubber emits 2.75 kg CO_2/kg material, while natural rubber and uncoated flat glass emit 2.02 and 0.99 kg CO_2/kg.

Other potentially important materials specific to EV batteries may be cobalt, nickel and lithium, which are not considered in our model. Comparing our inventory and GHG emission results to that of a recently published and very detailed study [18], we roughly estimate that the error of leaving out these materials is on the order of 15% for the battery alone. This translates into an error of about 6% for the production of the vehicle as a whole, and an error of merely 1% for the entire EV life cycle including charging electricity in the model's base year. We acknowledge that this error could be higher in future years assuming a decarbonization of the electricity mix. However, simultaneous improvements in battery technology would be conceivable as well, so that these effects could cancel each other out. We therefore anticipate that including the embodied CO_2 emissions from cobalt, nickel and lithium would not notably change scenario outcomes.

Further, our single–region LCA model assumes that vehicle production takes place in the US. In reality, many of the

vehicles bought in the US are made elsewhere in the world. On the other hand, quite a few are exported.[1] We present evidence that the error invoked from this assumption is small however. To that end we analyze the differences between the 'full pricing' and the 'well-to-wheel' pricing scenario. The 'full pricing' scenario fully prices embodied emissions of vehicle and battery production while the 'well–to–wheel pricing' scenario excludes pricing of vehicle and battery production emissions altogether. Yet, the differences in sales are marginal: merely a few longer–range BEVs are partially replaced by shorter–range BEVs (Supplementary Figure 6h). Furthermore, these sales differences do not notably affect overall emissions outcomes (Figure 2a). As documented in Supplementary Table 2, the difference in total fleet–wide life cycle emissions between the two scenarios amounts to 71 Mt CO_2 cumulatively over the period 2010–2050. Hence, the results of this study are fairly unsusceptible to assumptions regarding the carbon intensity of vehicle and battery production which in turn partly depend on the location of production (inside versus outside of the US).

Supplemental References

[1] Paul Wolfram, Qingshi Tu, Niko Heeren, Stefan Pauliuk, and Edgar G Hertwich. Material efficiency and climate change mitigation of passenger vehicles. *Journal of Industrial Ecology*, 25(2):494–510, 2021.

[2] P. Wolfram, Q. Tu, E. Hertwich, and S. Pauliuk. Documentation of the transport-sector model within the RECC model framework. Zenodo, 2020.

[3] Mohammad S Masnadi, Hassan M El-Houjeiri, Dominik Schunack, Yunpo Li, Jacob G Englander, Alhassan Badahdah, Jean-Christophe Monfort, James E Anderson, Timothy J Wallington, Joule A Bergerson, et al. Global carbon intensity of crude oil production. *Science*, 361(6405):851–853, 2018.

[4] Liang Jing, Hassan M El-Houjeiri, Jean-Christophe Monfort, Adam R Brandt, Mohammad S Masnadi, Deborah Gordon, and Joule A Bergerson. Carbon intensity of global crude oil refining and mitigation potential. *Nature Climate Change*, 10(6):526–532, 2020.

[5] Cristina Antonini, Karin Treyer, Anne Streb, Mijndert van der Spek, Christian Bauer, and Marco Mazzotti. Hydrogen production from natural gas and biomethane with carbon capture and storage–a techno-environmental analysis. *Sustainable Energy & Fuels*, 4(6):2967–2986, 2020.

[6] Robert Edwards, Heinz Hass, Jean-Francois Larive, Laura Lonza, Heiko Maas, and David Rickeard. Future automotive fuels and powertrains in the European context – version 4.a, 2014.

[7] Linda Ager-Wick Ellingsen, Bhawna Singh, and Anders Hammer Strømman. The size and range effect: Lifecycle greenhouse gas emissions of electric vehicles. *Environmental Research Letters*, 11(5):054010, 2016.

[8] Troy R. Hawkins, Bhawna Singh, Guillaume Majeau-Bettez, and Anders Hammer Strømman. Comparative Environmental Life Cycle Assessment of Conventional and Electric Vehicles. *Journal of Industrial Ecology*, 17(1):53–64, 2012.

[9] S. Lu. Vehicle survivability and travel mileage schedules. US Department of Transportation, National Highway Traffic Safety Association, Washington DC., 2006.

[10] Paul Wolfram and Nic Lutsey. Electric vehicles: Literature review of technology costs and carbon emissions. International Council on Clean Transportation, 2016.

[11] O. Y. Edelenbosch, A. F. Hof, B. Nykvist, B. Girod, and D. P. van Vuuren. Transport electrification: the effect of recent battery cost reduction on future emission scenarios. *Climatic Change*, 151(2):95–108, Sep 2018.

[12] Nic Lutsey and Michael Nicholas. Update on electric vehicle costs in the United States through 2030. International Council on Clean Transportation, 2019.

[13] IRENA. Future of solar photovoltaic: Deployment, investment, technology, grid integration and socio-economic aspects (a global energy transformation: paper). International Renewable Energy Agency, Abu Dhabi, 2019.

[14] IRENA. Future of wind: Deployment, investment, technology, grid integration and socio-economic aspects (a global energy transformation: paper). International Renewable Energy Agency, Abu Dhabi, 2019.

[1] https://legacy.trade.gov/td/otm/autostats.asp

[15] Paul Wolfram and Edgar Hertwich. Representing vehicle-technological opportunities in integrated energy modeling. *Transportation Research Part D: Transport and Environment*, 73:76–86, 2019.

[16] A. Burnham, M. Wang, and Y. Wu. Development and applications of GREET 2.7 — the transportation vehicle-cycle model. Energy Systems Division, Argonne National Laboratory, 2006.

[17] Gregor Wernet, Christian Bauer, Bernhard Steubing, Jürgen Reinhard, Emilia Moreno-Ruiz, and Bo Weidema. The ecoinvent database version 3 (part I): overview and methodology. *The International Journal of Life Cycle Assessment*, 21(9):1218–1230, 2016.

[18] Jarod C Kelly, Qiang Dai, and Michael Wang. Globally regional life cycle analysis of automotive lithium-ion nickel manganese cobalt batteries. *Mitigation and Adaptation Strategies for Global Change*, 25(3):371–396, 2020.
