# Supplementary material for: Pricing indirect emissions accelerates low—carbon transition of US light vehicle sector
Source: Nat Commun. 2021 Dec 8;12:7121. doi: 10.1038/s41467-021-27247-y (PMC8654946; doi:10.1038/s41467-021-27247-y)
Supplement: Supplementary file 4 — Description of Additional Supplementary Information [file 41467_2021_27247_MOESM4_ESM.pdf]

Description of Additional Supplementary Files

File Name: Supplementary Data 1

Description: This electronic supplementary material provides the most important input data and all results presented in Wolfram et al. (2021) "Pricing indirect emissions accelerates low-carbon transition of US light vehicle sector", Nature Communications.
